# Waddington, Dynamic Systems, and Epigenetics

**DOI:** 10.3389/fnbeh.2016.00107

**Published:** 2016-06-10

**Authors:** Ed Tronick, Richard G. Hunter

**Affiliations:** Developmental and Brain Sciences, University of Massachusetts Boston, PsychologyBoston, MA, USA

**Keywords:** gene environment interactions, biological, adaptation, psychological, behavioral development, animal models, human development, social environment

## Abstract

Waddington coined the term “epigenetic” to attempt to explain the complex, dynamic interactions between the developmental environment and the genome that led to the production of phenotype. Waddington's thoughts on the importance of both adaptability and canalization of phenotypic development are worth recalling as well, as they emphasize the available range for epigenetic action and the importance of environmental feedback (or lack thereof) in the development of complex traits. We suggest that a dynamic systems view fits well with Waddington's conception of epigenetics in the developmental context, as well as shedding light on the study of the molecular epigenetic effects of the environment on brain and behavior. Further, the dynamic systems view emphasizes the importance of the multi-directional interchange between the organism, the genome and various aspects of the environment to the ultimate phenotype.

Waddington introduced the term epigenetics in 1942 (Waddington, 1942) as a refinement of his conception of an “epigenetic landscape” (Waddington, 1940). He used the term to describe the class of internal and external interactions between the environment and the genes leading to the development of phenotype. By the end of the 1950's the term had become, in Lederberg's view “a semantic morass” (Lederberg, 2001). This remains true to this day. Recent attempts to codify a consensus definition are more molecular. The definition of “mitotically and/or meiotically heritable changes in gene function that cannot be explained by changes in DNA sequence” (Russo et al., 1996) is close to a consensus, though the term is also used to describe phenomena that utilize molecular epigenetic mechanisms (histone modifications, or DNA methylation) which merely alter the transcriptional phenotype of a cell (Griffiths and Hunter, 2014). This paper is an attempt to give our own perspective as a developmentalist and a stress neurobiologist on epigenetics in the broader context as we see it (Lester et al., 2011; Griffiths and Hunter, 2014; Hunter, 2014).

In molecular epigenetics the term “epi” is interpreted as meaning “over,” as in the molecular process sitting over and operating on the genes; However, Waddington knew nothing about molecular processes as sitting over the genes, Avery's identification of DNA as the genetic material wasn't published until 1944 (Avery et al., 1944) and Waddington could only theorize about the processes involved. His theoretical work was of a piece with his experimental work on environmental influences on the development of phenotype in Drosophila [see (Robertson, 1977) an excellent overview of Waddington's life and work], His view was that there was a landscape of choices facing an organism and the initial constraints and starting point were set by genes, but during development environmental and physiologic forces, increasingly came into play. These forces would then operate along with, and in interaction with genes and each other over time and push (structure) the organism into typically deeper canals resulting in the organism's eventual phenotype. The interactive process—canalization—meant that individual organisms that might have identical genetic make-up could develop radically different phenotypes (Waddington, 1957). His view, perhaps predated in some ways by Lamarck [though Waddington wasn't a Lamarckian (Waddington, 1957)], was an initial clear statement of a mechanistic theory of gene X environment (GxE) interaction.

His conceptualization had profound influences on different fields, especially developmental fields, which strive to specify the nature of the environment and its underlying physiologic and later neurophysiologic effects in interaction with genes on the eventual phenotype of the organism. While the search has been fruitful, it has always left many thinking that the GxE connection was often more in our conceptualizations than in biologic reality. The question was always, how the environment modified gene function in lasting, “canalized,” ways. Regulation of transcription was a potential solution to the problem. Nonetheless it was unclear how the persistent changes in global patterns of gene expression were orchestrated by discrete environmental events (e.g., early life trauma, toxic exposure), or by less clearly delimited inputs and more continuous inputs, such as parental behavior.

Molecular epigenetics has changed all that with its sitting-on-top-of-role operating on genes and its openness to influences from the surrounding environment. Waddington would have loved the discovery but perhaps not as much as we love it. After all he was already committed to the multiplicity of environmental factors along with dynamic changes in the organism as the organism became more organized—canalized. But maybe not, since love blinds us to many things. Nonetheless we will to try to cut away the blinders just a bit. In so doing we will be building not only on the work of Waddington, but of many others, notably Gottlieb (1995), and attempt to introduce complications of our own.

Waddington's epigenetics, like molecular epigenetics is was anchored in evolutionary theory. Nonetheless, molecular epigenetics govern shorter term ontogenetic processes that can operate on a fast time scale (even minutes) to ontogenetic time (a lifetime), and likely, for some even, intergenerationally. Indeed one way to see the functional role of molecular epigenetics is that it is a way for the organism to make “quick” adaptations to the changing circumstances. Indeed, Waddington spent much ink arguing that “adaptability” was an important adaptation in-and-of-itself and that it was epigenetic in nature (Waddington, 1957). The example of fetal programming is just such a model where in utero information leads to fetal organization based on the information it is receiving. But other mechanisms, learning in particular serve the same purpose and may or may not involve molecular epigenetic processes. Regardless, such changes may or may not turn out to be adaptive in the evolutionary sense of the term. Meaney's research on maternal licking and grooming (LG) in rodents is a postnatal model of such changes (Weaver et al., 2004; Meaney et al., 2007; Turecki and Meaney, 2016). Though many see the high LG animals as more adapted, especially work on human parenting where high LG equates to sensitive parenting, we, along with Meaney, argue that high LG or low LG is only “adaptive” in the context the animal has to engage. The capacity of rats to show this sort of flexibility in behavioral phenotype, without changes in underlying genotype, is a perfect example of Waddington's conception of epigenetics as trait adaptability.

Waddington's idea of canalization appears closer to the classical genetic view of development, but for him it was complementary to adaptability (Waddington, 1957). As the organism became more endogenously structured it typically found itself in deeper pathways—canals—that further constrained change over time; the eventual phenotype became more predictable. Moreover, genetic control of the phenotype has the advantage (and disadvantage) of being based on a longer evolutionary history of experience in the environment, which changes on different, often faster time scale. A more canalized, route from genotype to phenotype is often superior to an adaptable one in terms of survival, particularly when the precise function of a particular gene product is necessary for the function of larger gene networks. The large number of embryonic lethal mutations [roughly a third of gene knock outs in mice (Dickerson et al., 2011)] speaks to the importance of canalization for successful development. The fact that most gene knock-outs are not lethal gives an idea of the outer bound on “adaptability,” in mice at least.

A more contemporary statement can be framed in dynamic systems theory with its notions of complexity, states, and the dynamics of change. Over recent decades much of biology has moved from a static view of biological structure and function to a dynamic view that follows from an understanding of biochemical mechanism. All biologic systems are open dynamic systems that must successfully gain energy and information to maintain their organization and to develop; in the face of possible dissipation when they are unsuccessful. Thus open, living systems are always continuously engaged with the environment. Furthermore, they are complex in the sense that they are made up of hierarchically organized multiple parts serving different functions. They also operate with multiple feedback and feedforward loops which limit or amplify the effects of external or internal processes. Causality is complex and often appears circular and effects that emerge at one point in time become part of the organism's on-going processes or organization that affect what happens at the next point in time. It is a dynamic that can only be seen over a variety of different time scales at different hierarchical levels with multiple measures of different processes.

These characteristics are close to Waddington's notions of canalization of the increasing differentiation and structuring of the organism. Closer still is the notion of a state space; the landscape of states, the momentary to more long-lasting organization an organism can be in. Attractor states, are deep canals that are hard to leave, like the mature phenotype, whereas other states are shallow troughs, easy to get into and out of, like the early organization of the developing phenotype. The operation of the autonomic nervous system with its fight or flight states, is one example of organismic states. Another is inflammatory immune processes with state effects in body and brain from the molecular to the behavioral (Haroon et al., 2012).

How does this dynamic systems perspective help us remove some of the blinders we have about molecular epigenetics? A dynamic systems perspective has as a first principle that that organism is always engaged with the environment. The principle demands careful characterization of the environment and the organism's engagement with it. Despite epigenetics' critical recognition that the environment affects epigenetic mechanisms, the characterization of the environment is often underspecified especially compared to the elegance of the molecular work. Typical studies looking at epigenetic changes may utilize a well-characterized experimental manipulation of the environment, only to return the animals to an unobserved environment for periods of time that may even go over developmental transitions and sensitive periods until the change is assessed.

Even in the best animal experimental studies, factors in addition to the study proper, such as housing conditions, events during animal housing, handling regimes, light cycles, social contacts, etc., are not well-specified. The “environmental phenotyping” is crude. Such a lack of concern can easily lead to false conclusions. In particular, the conclusion that the initially induced epigenetic change is stable—fixed—whereas the observed stability may be the product of or maintained by the ongoing environment; indeed even more radically the epigenetic change might not be stable in-and-of-itself but repeatedly instantiated—created anew—by the ongoing environment. This is especially important when epigenetic changes are induced by a stressful manipulation and the intervening unobserved environment is also stressful, which likely characterizes many features of animals' housing environments (Crabbe et al., 1999).

It is established that enriched environments are protective against the effects of stress upon the brain in laboratory animals (Mora et al., 2012; Crofton et al., 2015). Less well-established is whether normal housing is really a neutral environment or a stressor unto itself relative to the “natural” environment of laboratory animals, if such can be said to exist. In humans, early trauma events may be documented with associated epigenetic changes, but the unobserved environment could easily maintain any changes or induce others because traumatic environments often are chronically stressful, even toxically stressful. This lack of concern flies in the face of Waddington's view and what we know about the many factors that will lead to dynamic systems change. Without a careful characterization of the environment, changes ascribed to molecular epigenetic changes may in the end, turn out to be *epi (false)*-phenomenon. What we think is needed is as much effort into characterizing the details of the experience of the animal or human and its environment as has gone into characterizing molecular mechanisms.

A critical dynamic issue is how the environmental manipulation and its associated epigenetic change changes the animal's engagement with the environment. The presumption in epigenetics is that epigenetic changes are associated with a host of endogenous changes at multiple levels. That is the way systems operate. A change at one or another level of a system changes other levels such that the animal under study is not the same as it was after the change. Even in a perfectly characterized unchanging environment, what the animal does in the environment after a manipulation will change, and these changes in engagement could or more likely will have additional epigenetic effects. Moreover, the animal that moves into a different state of organization because of an epigenetic change not only engages that environment in a different fashion, but actually changes the environment and itself in an on-going fashion. This is Dawkins' idea of the extended phenotype (Dawkins, 1978). The initial change becomes part of an on-going process of change. That is, epigenetic changes related specifically to external environmental events in turn become causal elements that go on to amplify, stabilize, or inhibit other epigenetic changes.

These systemic dynamics have come to be appreciated in studies of physiologic systems such as the HPA axis, where physiologic and behavioral feedback and feed-forward loops operating over time are critical to understanding how the organism functions. To adapt a metaphor, which may help in seeing the system dynamics of this process: rain drops—environmental changes—sculpt a landscape—the organization of the animal including epigenetic changes. The new sculpted pathways—the state space the animal comes to occupy—constrains where the rain can flow—the animal engages the changes in a new way, yet at the same time the pathways—the organization of the animal—continue to be shaped by the rain—environmental changes, such that even a repeated same event does not produce the same effect. Thus not only do we need detailed studies of the multilevel pathways changed by an environmental manipulation, we must study the dynamics of the continuous engagement of the organism with the environment over time.

Much of what we know about epigenetic changes, especially in humans, is from studies of models of abnormal processes, such as toxic exposures, deprivation or experimental paradigms. These studies are without doubt revealing but they may not characterize the typical operation of epigenetic processes. From a dynamic systems perspective, while some animals exist in what may be thought of as outlier locales in the species' typical state space, how they are functioning may not characterize the operation of the more typical organism. They are almost by definition a-typical. Thus, they may be poor models of typical processes, but critically, we can only know what that is if we know what the typical operation looks like. Fields concerned with development of organisms are quite aware of this, but the idea of needing to know what is “normal” has not yet been incorporated into epigenetic research. Indeed, much of the behavioral and developmental epigenetics have emerged from organisms adapted to the unusual environment of the modern laboratory. As such many basic questions with regard to the molecular epigenetics of developmental sensitive or critical periods are largely unexplored (Lester et al., 2011; Barr and Misener, 2016). Moreover, and critically the induction of changes is more than likely related to quotidian processes rather than extreme events. Waddington laid out a similar structure with his epigenetic landscape, where both environmental events and the genetic program of development opened shallow valleys when the track of development might change, and sharp defiles where these forces act to prevent any divergence from a particular, canalized outcome (Waddington, 1940). These dynamics, if studied would have profound effects for identifying the events and their timing that trigger the epigenetic mechanisms (e.g., methylation, the release from methylation, acetylation etc.). But at the moment much of what we know may be related to aberrant processes that may fall outside the range and dynamics of normal epigenetic processes and typical experience in typical environments. Given that, we need to engage in studies of more normative processes.

It would seem clear to many that the state of art in animal research is in advance of the work on humans. The most likely reasons are the control, albeit often imperfect, that animal researchers have over the experience of the animals and their ability to evaluate brain changes. Meaney's work with the detailed, multilevel characterization of mother infant interaction in rodents stands out as an example of how animal work can be done well. However, the difference should not be exaggerated. Developmentalists and other behavioral scientists have a long history of careful characterization of the environment and patterns of engagement of the individual with that environment. This characterization occurs at many temporal levels, but of special note is the engagement of individuals at micro-temporal levels (e.g., seconds, minutes) over time. With the exception of work on non-human primates, few molecular epigenetic studies characterize animals' forms of engagement with the environment, particularly the social environment, with equal quality to that of developmentalists. Moreover, developmentalists have a deep concern for longitudinal studies, studies that are sorely lacking in recent animal work.

As to the lack of access to brain tissue, human work will always lag. Nonetheless human studies have non-invasive ways of examining brain function in the engaged individual. These techniques may reveal equivalently valuable information as do neuroanatomical studies of sacrificed animals. Also, human studies often employ measure of other systems, the autonomic nervous system, the immune system, the cardiac system, the neuroendocrine system. These approaches are possible in animal systems, but are not often pursued for methodological and economic reasons, as well as disciplinary canalization. Though not necessarily at the molecular level, the evaluation of these systems exposes underlying mediating processes or proximal pathways which are systems that affect molecular epigenetic changes. Indeed from a dynamic systems perspective, making brain tissue examination the gold standard along with molecular processes may be mistaken since the outcome of any manipulation is the result of multilevel interactions over time. Nonetheless, given the centrality of the brain to the developing organism's interaction with the environment, work on this organ is of substantial importance if it can be done with an equally rigorous eye to the environment. Animal studies will be important in this regard, both due to the ability to make observations at the molecular level in brain tissue and the ability to control the developmental environment in detail. While not flashy, there is a real need for descriptive studies of the brain epigenome during normal development as well as rigorous studies of how specific environmental interventions influence a broad range of molecular epigenetic marks in the brain.

We utilize the two models (see Figures 1, 2) to guide our work on humans and animals, especially social and altricial animals (DiCorcia and Tronick, 2011; Conradt et al., 2016; Liu and Tronick, 2016). In Figure 1 on the left are some of, but hardly all of the factors that affect physical and mental functioning to the right. In between are two distinct areas. The box at the bottom suggests some of the processes (e.g., immune, metabolic) that mediate the effects of the factors on the left as they affect health outcomes. In the center is caretaker-offspring interactive system that in humans and many other mammals regulates the state of the infant across developmental time. In this model the caretaker-offspring system can either buffer the offspring from the effects of factors on the left or transduce the effects of those factors to the offspring. The quality of the buffering or transduction during development has a powerful effect on underlying mediating processes, including epigenetic processes. The two directional nature of the arrows is an attempt to embed the idea that there are effects within each of the domains and among the domains that create complicated feedback and feedforward loops of amplification and regulation. Figure 2 attempts to capture the idea that these processes operate over time and can only be understood by looking at them over time. Yet it is necessarily incomplete due to our incomplete knowledge of the processes and interactions involved.

**Figure 1:**
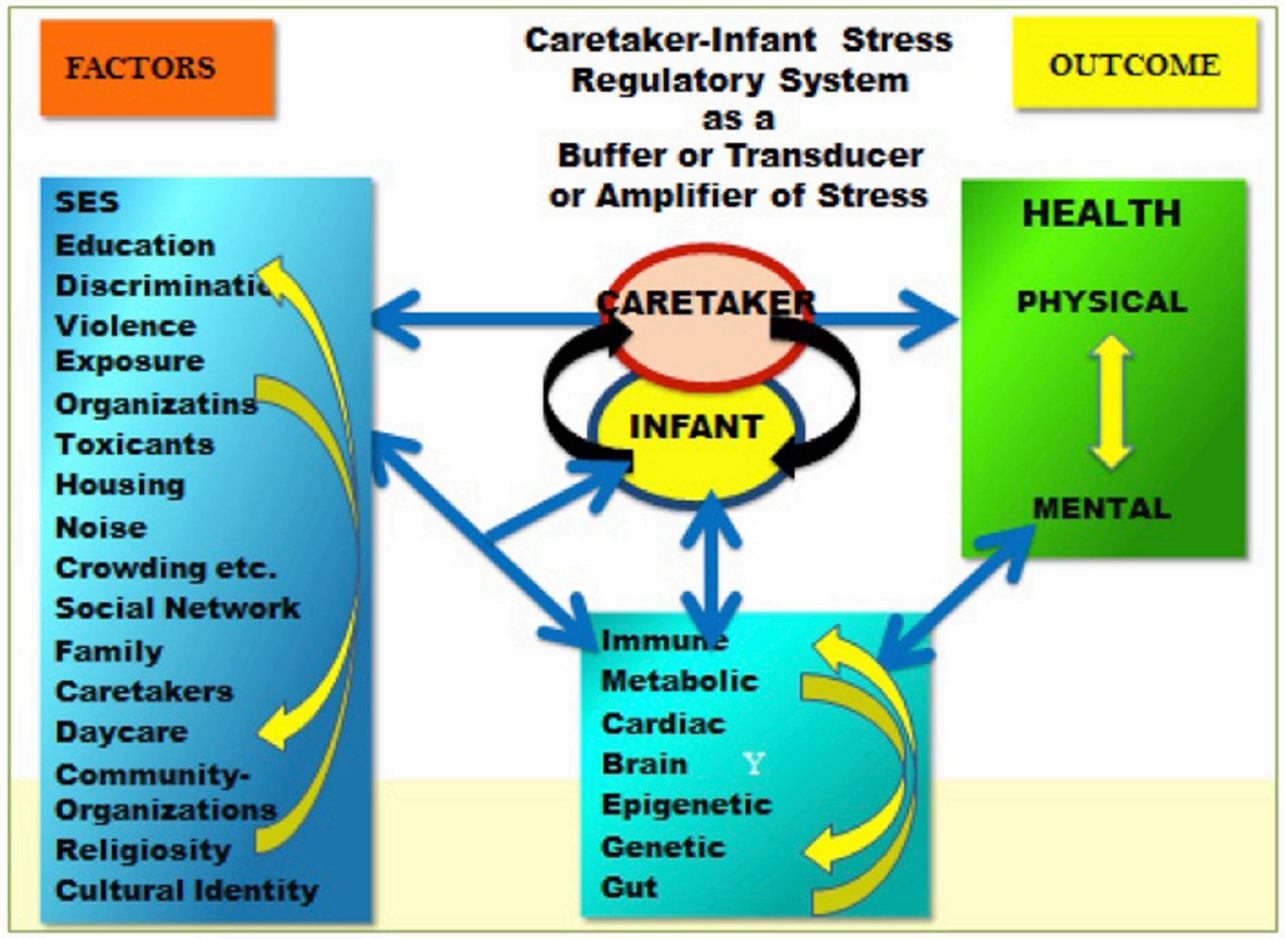
**On the left are some of the factors that affect physical and mental functioning to the right**. In between are two distinct areas. The box at the bottom suggests some of the processes that mediate the effects of the factors on the left as they affect health outcomes. The figure in the middle is the caretaker offspring interactive system that in humans and many other mammals regulates the state of the infant. In this model the caretaker-offspring system can either buffer the offspring from the effects of factors on the left or transduce the effects of those factors to the offspring.

**Figure 2:**
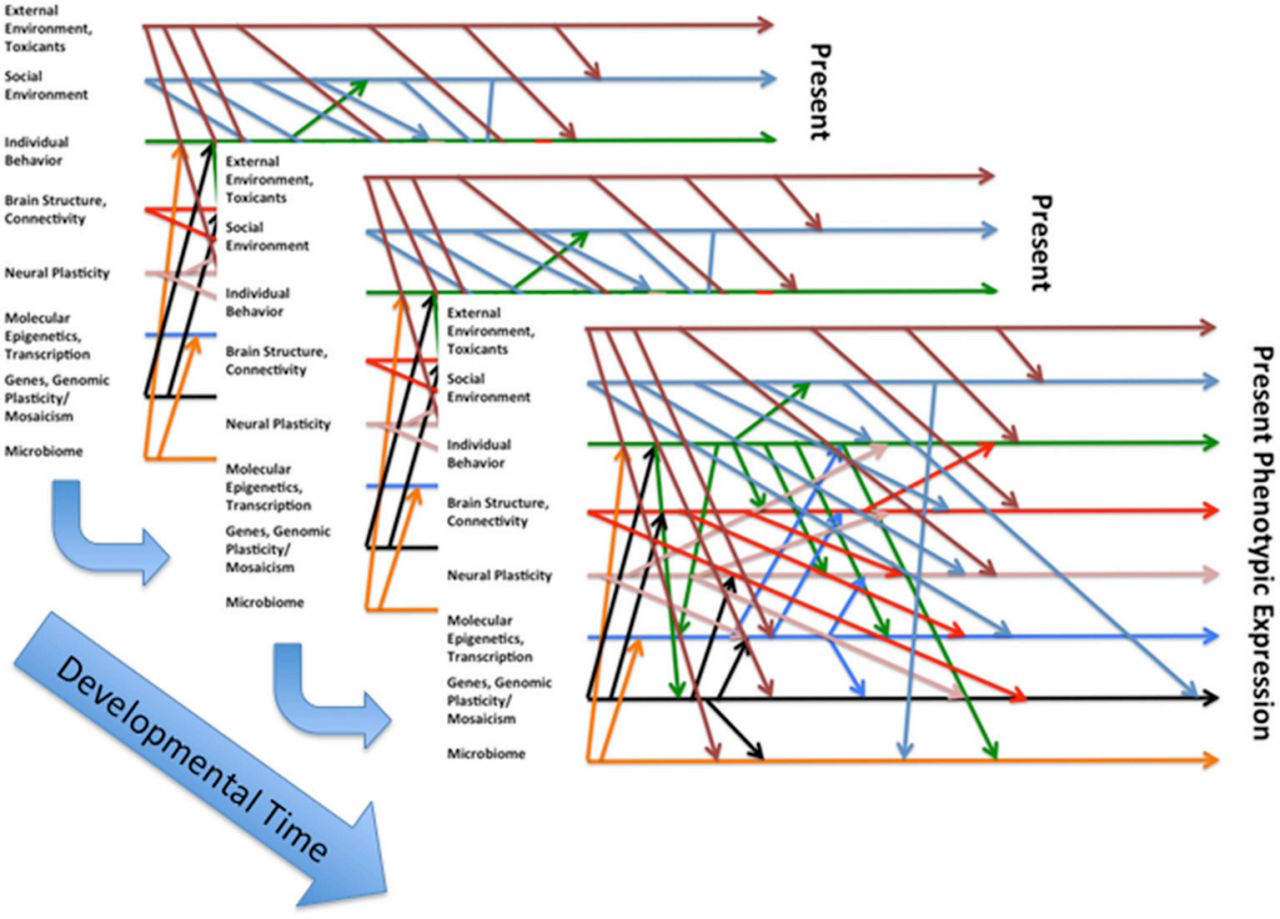
**An attempt to define some of the interactions that lead to a particular expressed phenotype at a particular point in developmental time, a nod to Gottlieb's (Waddington, 1957) somewhat more simplified conception of the interactions between different layers of causality with regard to the developmental evolution of phenotype**.

It should be clear that these dynamic models are far more complex and demanding than could be enacted in a single study. Certainly we have not done it even in our studies, in which we have looked at epigenetic changes in relation to caretaking and other factors longitudinally. At best we only gain traction on some components and processes of the models and it is already simplified. However, the reason for presenting them is to have them serve as a cautionary note. While the epigenetic studies in this Journal and elsewhere are formidable, we must not let them lead us into simplified thinking. Even if simplification is a necessary part keeping the dynamic complexity in mind, in our conceptualizations will make it far more likely will not miss things and increases the likelihood we will grapple with and uncover phenomenon of significance. While being thrilled with what we are finding out about with molecular epigenetics, we need to remove our blinkers, or at least acknowledge we are wearing them. We need to think as Waddington might have thought, with an eye to the broader context of living systems in an environment with both characterized by complexity and change. Thus while we can't do empirical justice to dynamic nature of the systems we are studying, and we can only write about what we have found in our studies, we can nonetheless keep in mind and speak of their complexity, even if it is to only remind ourselves of the unstudied complexity, as well as the complexity we could study, but have thus far set aside.

## Author contributions

Both authors (ET and RH) wrote and conceived of this perspective.

## Funding

Dr. ET acknowledges the support of the NIH: 1 R01 HD083267-01 and NSF 1457111.

### Conflict of interest statement

The authors declare that the research was conducted in the absence of any commercial or financial relationships that could be construed as a potential conflict of interest.
